# JBO 2022 List of Reviewers

**DOI:** 10.1117/1.JBO.28.1.010102

**Published:** 2023-01-12

## Abstract

Thanks to reviewers who served JBO in 2022.

The *Journal of Biomedical Optics* sincerely thanks the following individuals who served as reviewers in 2022. The success of our publication hinges on the voluntary contributions of time and energy put forth by these professionals.

Atefeh Abdolmanafi
Alireza Akbarzadeh
Walter Akers
Alba Alfonso-Garcia
Anna Letizia Allegra Mascaro
Marcello Amaral
Arjen Amelink
Alexander Antaris
Bahman Anvari
Matthew Applegate
Praveen Arany
Kamran Avanaki
Timothy Baran
Giovanni Barbera
Margarida Barroso
Connor Barth
Adam Bauer
Muyinatu Bell
Mikhail Berezin
Edouard Berrocal
Renzhe Bi
Thomas Bischof
Richard Blackmon
Matthieu Boffety
Nienke Bosschaart
Audrey Bowden
Adrian Bradu
J. Quincy Brown
Maruthi Manoj Brundavanam
David Busch
Alexander Bykov
Liangcai Cao
Xu Cao
Jeffrey Cassidy
Jonathan Celli
Shuang Chang
Qing Chao
Defu Chen
Nanguang Chen
Ata Chizari
Bernard Choi
Krzysztof Chrzanowski
Joseph Chue-Sang
Euiheon Chung
Kenny Chung
Samuel Chung
Sohyun Chung
Andrea Curatolo
Anabela Da Silva
Frederick Dallaire
Michael Daly
Arash Darafsheh
Roberto De Blasi
Luis De Taboada
Dinesh Dhankhar
Luca Di Cecilia
Jouke Dijkstra
Yichen Ding
Irina Dolganova
Chen-Yuan Dong
Alexander Doronin
Tatjana Dostalova
Alexandre Douplik
Arnaud Dubois
Andrey Dunaev
Nicholas Durr
Daniel Elson
Sefik Evren Erdener
Olga Eremina
Thomas Ertl
Dirk Faber
Chris Fadden
Andrea Farina
Hamid Farrokhi
Baowei Fei
Peng Fei
Jinchao Feng
Pietro Ferraro
Jeffrey Folz
Mitsuhiro (Hiro) Fukuda
Stephanie Gallant
Fei Gao
Shan Gao
Missael Garcia
Daniel Gareau
Yuval Garini
Jerome Gateau
Arkadiusz Gertych
Anuradha Godavarty
Dimitris Gorpas
David Orlando Grajales Lopera
Huanqing Guo
Xinxin Guo
Zeinab Hajjarian
Takafumi Hamaoka
Katharine Hanlon
Carole Hayakawa
Sicong He
Tiffany Heaster
Christine Hendon
Brian Hill
Jason Hirshburg
Pauliina Hirvi
Vladimir Hovhannisyan
Dewei Hu
Eno Hysi
Justus Ilgner
Xavier Intes
Seva Ioussoufovitch
Minbiao Ji
Mengyu Jia
Huabei Jiang
Chulmin Joo
Dongkyun Kang
Kavon Karrobi
Deepa Kasaragod
Masamoto Kazuto
Matthew Keller
Gordon Kennedy
Anjul Khadria
Alexander Khmaladze
Chulhong Kim
Jeesu Kim
Mikhail Kirillin
Tiffany Ko
Sanathana Konugolu Venkata Sekar
Ivan Kosik
Sri-Rajasekhar Kothapalli
Tess Kramer
Grey Kuling
Vladimir Kuzmin
Xiaomin Lai
Jesse Lam
Frederic Lange
Kirill Larin
Ethan LaRochelle
Bahman Lashkari
Lawrence Le
V. N. Du Le
Martin Leahy
Hee Ryung Lee
Kijoon Lee
Robert Lee
Paul Lemaillet
Frederic Lesage
Ofer Levi
Andrew Levy
Dongyu Li
Jiao Li
Lei Li
Peng Li
Qingli Li
Shuying Li
Ting Li
Weizhi Li
Xingde Li
Yingguang Li
Rongguang Liang
Maritoni Litorja
Chengbo Liu
Gangjun Liu
Liwei Liu
Quan Liu
Jonathan Lovell
Guolan Lu
Rongwen Lu
Geoffrey Luke
Jianwen Luo
Zhenhe Ma
Philipp Machler
Caroline Magnain
Shuichi Makita
Francesca Manni
Zbignevs Marcinkevics
Oszust Mariusz
Haley Marks
Igor Meglinski
Marcello Melis
Guanghan Meng
Zhaokai Meng
Arcangelo Merla
J. Sven Mieog
Takeo Minamikawa
Guillermo Monroy
Bruno Montcel
Mohesh Moothanchery
Sakina Mohammed Mota
Peter Munro
Peter Naglic
Achuth Nair
Sreyankar Nandy
Sujatha Narayanan Unni
Meda Negrutiu
Nozomi Nishimura
Navid Ibtehaj Nizam
Farouk Nouizi
Tatiana Novikova
Robert Nuster
Thomas O’Sullivan
Marien Ochoa Mendoza
Paul Okagbare
Chukwuemeka Okoro
Jeffrey Oliver
Xavier Orlik
Antonio Ortega-Martinez
Rahul Pal
Gregory Palmer
Vikas Pandey
Vimal Prabhu Pandiyan
Arturo Pardo
Junha Park
Soongho Park
Prasant Pattnaik
Nicholas Pereira
Hien Thi-Thu Pham
Thao Pham
Shaohua Pi
Daqing Piao
Mark Pierce
Anahita Pilvar
Arthur Plante
Raju Poddar
Adrian Podoleanu
Brian Pogue
Jenni Poimala
Alexey Popov
Anouk Post
Scott Prahl
Jaya Prakash
Le Qiu
Matin Raayai Ardakani
Praveenbalaji Rajendran
Jessica Ramella-Roman
Lise Randeberg
Nathaniel Redgewell
Samuel Remedios
Jose Rico-Jimenez
Cody Rounds
Alberto Ruiz
H. Grady Rylander III
Marcelo Saito Nogueira
Inga Saknite
Ahmad Salman
Kimberley Samkoe
Angelo Sassaroli
Travis Sawyer
Iwan Schie
Felix Scholkmann
Fynn Schwiegelshohn
Janaka Senarathna
Ikbal Sencan-Egilmez
Babak Shadgan
Rubina Shaikh
Yu Shang
Anna Sharikova
Guillaume Sheehy
Shuhao Shen
Sherif Sherif
Benjamin Sherlock
Lingyan Shi
Leonid Shmuylovich
Ke Si
Lu Si
Koneswaran Sivagurunathan
Jason Smith
Zachary Smith
Janis Spigulis
Radhakrishnan Srinivasan
Vishal Srivastava
Ronald Sroka
Samuel Streeter
Tomas Stromberg
Eric Suero Molina
Ulas Sunar
Johannes Swartling
Syeda Tabassum
Enrique Tajahuerce
Bingyao Tan
Tanja Tarvainen
Dieter Richard Taubert
Lioudmila Tchvialeva
Jeffrey Thatcher
Chao Tian
Jie Tian
Zhiqiang Tian
Kenneth Tichauer
Vassiliy Tsytsarev
Valery Tuchin
Ioan Turcu
Giovanni Ughi
Pablo Valdes
Martin van Gemert
Gijs Van Soest
Jenu Varghese Chacko
Sarah Vavrek
Umberto Villa
Karthik Vishwanath
Heidrun Wabnitz
Alexandra Walsh
Lei Wang
Lidai Wang
Lin Wang
Adam Wax
Timothy Weber
Robert Weersink
Jesse Wilson
Terence Wong
Michael Wood
Jiamin Wu
Min Wu
Rosalind Wynne
Lei Xi
Jun Xia
Liangzhong Xiang
Weisi Xie
Chen Xu
Guan Xu
Ping Xue
Alexander Yakunin
Phaneendra Yalavarthy
Shijie Yan
Bin Yang
Eric Yang
Guang Yang
Sihua Yang
Xinmai Yang
Xuefeng Yang
Irina Yanina
Gang Yao
Anna Yaroslavsky
Filiz Yesilkoy
Derrick Yong
Jonghee Yoon
Sixian You
Li Yu
Linhui Yu
Zhen Yuan
Vladimir Zaitsev
Jihad Zallat
Limin Zhang
Wei Zhang
Yide Zhang
Shuang Zhao
Yanyu Zhao
Chao Zhou
Hao Zhou
Xin Zhou
Caigang Zhu
Jiaqi Zhu
Liren Zhu
Shouping Zhu
Lawrence Ziegler

